# SLC39A7 promotes malignant behaviors in glioma via the TNF-α-mediated NF-κB signaling pathway

**DOI:** 10.7150/jca.54158

**Published:** 2021-06-01

**Authors:** Lian Chen, Jinpeng Zhou, Long Li, Junshuang Zhao, Hao Li, Wei Zheng, Jinkun Xu, Zhitao Jing

**Affiliations:** 1 Department of Neurosurgery, the First Hospital of China Medical University, NO. 155 North Nanjing Street, Shenyang, 110001 China.; 2 Department of Histology and Embryology, College of Basic Medical Science, China Medical University, NO. 77 Puhe Road, Shenyang, 110122 China.

**Keywords:** SLC39A7, glioma, invasion, tumorigenesis, migration.

## Abstract

**Purpose:** Several studies have indicated that SLC39A7 plays an important role in tumor progression; however, little is known about the function and mechanism of SLC39A7 in glioma. In this study, we aimed to explore the role of SLC39A7 in glioma development.

**Patients and methods:** Bioinformatic analysis was used to predict the role of SLC39A7 in glioma. Cell viability and Edu assays were used to detect the proliferation of glioma cells. A transwell assay was used to measure the invasion and migration of glioma cells. Western blotting, qPCR and ELISA were used to detect the expression of all molecules.

**Results:** SLC39A7 was found to be highly expressed in high-grade glioma patients with a poor prognosis. Our results indicated that SLC39A7 significantly promoted the proliferation, invasion and migration of glioma cells. Furthermore, SLC39A7 promoted tumorigenesis in orthotopic models. We determined that SLC39A7 promotes the malignant behaviors of glioma by activating the TNF-α-mediated NF-κB signaling pathway.

**Conclusion:** Our study revealed that SLC39A7 promotes the proliferation, invasion and migration of glioma cells via the TNF-α-mediated NF-κB signaling pathway, which provides potential targets for glioma therapy.

## Introduction

Glioma is the most common primary malignant brain tumor in adults, accounting for about 30% of central nervous system tumors and 80% of brain tumors 1, 2. Despite the development of various comprehensive therapies, the therapeutic effects are still not satisfactory 3. The average overall survival of patients with glioblastoma (GBM), the most malignant type of glioma, remains approximately 12 to 15 months 4, 5. Therefore, in-depth studies of the mechanisms involved in molecular regulation and biological function in glioma progression are needed to develop more effective treatment strategies. Molecular therapy may provide reliable guidance for glioma therapy.

Zinc is essential for normal cell growth and development and is involved in protein, nucleic acid, carbohydrate and lipid metabolism, and the control of gene transcription, growth and differentiation 6, 7. Accumulating evidence indicates that zinc homeostasis disruption is related to cell malignant behaviors in human cancers 8, 9. One member of the zinc transporter family, solute carrier family 39, member 7 (SLC39A7), is predominately located at the endoplasmic reticulum (ER) membrane, the Golgi, or both 10. It has been shown to maintain zinc homeostasis through facilitating zinc influx into the cytoplasm from the ER. It was proven that SLC39A7 could promote intestinal epithelial self-renewal by resolving ER stress 11. SLC39A7 is phosphorylated by protein kinase casein CK2, associated with cell proliferation, migration, apoptosis, and mitosis 12, 13. Moreover, SLC39A7 has been reported to play a significant role in several cancers, such as cervical cancer, gastric cancer, and breast cancer 14-16. In gastric cancer, SLC39A7 induces gastric cancer cell proliferation and migration and inhibits apoptosis via the AKT/mTOR signaling pathway 15. However, the function and mechanism of SLC39A7 in glioma remains unclear.

In this study, we investigated the role and function of SLC39A7 in glioma. Our results indicated that SLC39A7 is highly expressed in glioma and is associated with a poor prognosis. Using bioinformatics analysis and *in vivo* and *in vitro* experiments, we found that SLC39A7 promotes glioma proliferation, invasion, migration and tumorigenesis via the TNFα-mediated NF-κB signaling pathway. This finding contributes to our understanding of SLC39A7 in glioma.

## Materials and Methods

### Patient samples and cell culture

This study was approved by the Ethics Committee of the First Affiliated Hospital of China Medical University, and written informed consent was obtained from each patient. The clinical samples were selected from 70 glioma patients from January 2007 to December 2012 at the First Affiliated Hospital of China Medical University. There were 20 samples of grade II, 25 samples of grade III and 25 samples of grade IV.

The human glioma cell lines, U87 and U251, were purchased from the Chinese Academy of Sciences cell bank (Shanghai, China). The human glioma cell lines, T98G and LN229, were purchased from the American Type Culture Collection (Manassas, VA, USA). The human glioma cell line H4 was purchased from iCell Bioscience (Shanghai, China). All glioma cell lines were maintained in Dulbecco's Modified Eagle's Medium (HyClone, Logan, UT, USA), supplemented with 10% fetal bovine serum (Gibco, Carlsbad, CA, USA) and 1% penicillin/streptomycin (Gibco) at 37 °C with 5% CO_2_. Normal human astrocytes were purchased from ScienCell Research Laboratories (San Diego, CA, USA) and maintained in an astrocyte medium (ScienCell Research Laboratories).

### Lentiviral vector construction and transfection

The lentivirus-based vectors for SLC39A7 overexpression, RNAi-mediated knockdown of SLC39A7, and its negative control were all constructed by Gene-Chem (Shanghai, China). The sequences of SLC39A7 siRNAs were as follows: KD1: 5'-CACCGCTCTCCCTCACCAGGCACTG-3' and KD2: 5'-CACCGAGCTGCTGAGATCAGCACTG-3'. Lentivirus transfection and efficacy measurements were performed as previously described 17.

### Real-time PCR

Real-time PCR was performed as previously described 17. The Mini-BEST Universal RNA Extraction kit (TaKaRa, Kyoto, Japan) was used to extract the total RNA of glioma cells. A Prime Script RT Master Mix reagent kit (TaKaRa) was used to synthesize cDNA. Finally, the qPCR assays were detected using the SYBR Green Master Mix (TaKaRa) with PCR LightCycler480 (Roche Diagnostics, Basel, Switzerland). β-actin was used as an endogenous control. The primers were: SLC39A7, forward: 5′-CTGGAGCGGTGAGAATGAGAGG-3′ and reverse: 5′- ACTGGTGGGAGAAAGGAAACTGG-3′; β-actin, forward: 5′-CATGTACGTTGCTATCCAGGC-3′, and reverse: 5′-CTCCTTAATGTCACGCACGAT-3′.

### Western blot analysis

Western blotting was performed as previously described 17. Briefly, the total proteins of glioma cells or tissues were isolated using a total cell protein extraction kit (KeyGen Biotechnology, Nanjing, China). Protein lysates were transferred onto PVDF membranes after electrophoresis and blocked with 2% bovine serum albumin (KeyGen Biotechnology). The primary antibodies against SLC39A7 (1:1000; Abcam), TNF-α (1:1000; Abcam), p-p65 (1:1000; Cell Signaling Technology, Danvers, MA, USA), p65 (1:1000; Cell Signaling Technology), p-IκBα (1:1000; Cell Signaling Technology), IκBα (1:1000; Cell Signaling Technology), p-IKKα/β (1:1000; Cell Signaling Technology), IKKα (1:500; Cell Signaling Technology), IKKβ (1:500; Cell Signaling Technology) and β-actin (1:2000; ProteinTech, Chicago, IL, USA) were incubated at 4 °C overnight. After secondary antibody (ProteinTech) incubation, the bands were detected using a chemiluminescence ECL kit (Beyotime Biotechnology, Beijing, China) and quantified by Image J software (National Institutes of Health, Bethesda, MD, USA).

### Immunohistochemistry (IHC)

IHC was performed as previously described 18. Briefly, the paraffin-embedded tissue sections were labeled with primary antibody against SLC39A7 (1:100; Abcam). Then, sections were treated with an immunohistochemical labeling kit (MaxVision Biotechnology, Fuzhou, China) and photographed with a light microscope (Olympus, Tokyo, Japan). The German immunohistochemical score was used to semi-quantify the expression 18, 19.

### Enzyme-linked immunosorbent (ELISA)

The commercially available ELISA kits (Cusabio, Stratech, UK) were used to detect the concentrations of TNF-α in the media supernatants of the glioma cells, as previously described 17.

### Cell viability assay

The glioma cells were plated in 96-well plates at a density of 1000 cells/well and cultured for 0, 24, 48, 72, 96 and 120 h. As previously described, cell viability was examined using the CellTiter 96® Aqueous Non-Radioactive Cell Proliferation Assay Kit (Promega, Madison, WI, USA) under the manufacturer's instructions.

### Edu assay

An Edu assay was performed as previously described 17 using an EdU assay kit (Beyotime, Biotechnology). Briefly, the glioma cells were seeded into 24-well plates at 1 × 10^5^ cells/well for 24 h, and 10 µM Edu reagent was added to the medium and incubated for 2 h. Then, cells were fixed, permeabilized and counterstained. The percentage of EdU-positive cells was calculated using a laser scanning confocal microscope (Olympus).

### Transwell invasion and migration assay

For the transwell assay, about 1 × 10^5^ glioma cells under different conditions were plated in the upper chamber (Corning) with (invasion) or without (migration) a Matrigel filter (BD), and DMEM containing 10% FBS was added to the lower chamber. After incubation for 24 h, the invading cells were fixed with 4% paraformaldehyde and stained with crystal violet (Beyotime Biotechnology). The stained cells were photographed and counted under a light microscope (Olympus).

### Xenograft experiments

Xenograft experiments were performed as previously described 18. Six-week-old female BALB/c nude mice (Beijing Vital River Laboratory Animal Technology, Beijing, China) were reared at the Laboratory Animal Center of China Medical University. Glioma cells under different conditions were injected orthotopically into the mouse brains at 5 × 10^4^ cells per mouse using a stereotaxic instrument (n = 5, per group). The tumor volume was measured according to the following formula: V = (D × d^2^) / 2, where D was the longest diameter and d was the shortest diameter of the tumor.

### Bioinformatic analysis

The data for mRNA expression, WHO grades, isocitrate dehydrogenase (IDH) status (IDH 1/2) of SLC39A7, the survival times and the status of glioma patients were obtained from the mRNA seq-325 dataset of the Chinese Glioma Genome Atlas (CGGA, http://www.cgga.org.cn), The Cancer Genome Atlas (TCGA, http://cancergenome.nih.gov) in the HG-U133A platform and Rembrandt databases. Gene set enrichment analysis (GSEA, http://www.broadinstitute.org/gsea/index.jsp) was used to analyze the enrichment of signal pathways between high and low SLC39A7 expression.

### Statistical analysis

Results are shown as the mean ± SD from at least three independent experiments. SPSS 23.0 software (IBM, Armonk, NY, USA) was employed for statistical analysis. The chi-square test and the two-tailed Student's t-test were used to compare the two groups' statistical significance. One-way analysis of variance was used to compare statistical significance among three or more groups. Pearson's correlation analysis was used to assess the correlation between the two groups. The survival difference was evaluated by a log-rank test and Kaplan-Meier analysis. Two-tailed P values < 0.05 were considered significant.

## Results

### SLC39A7 is highly expressed in gliomas and correlates with poor prognosis

**Figure 1:**
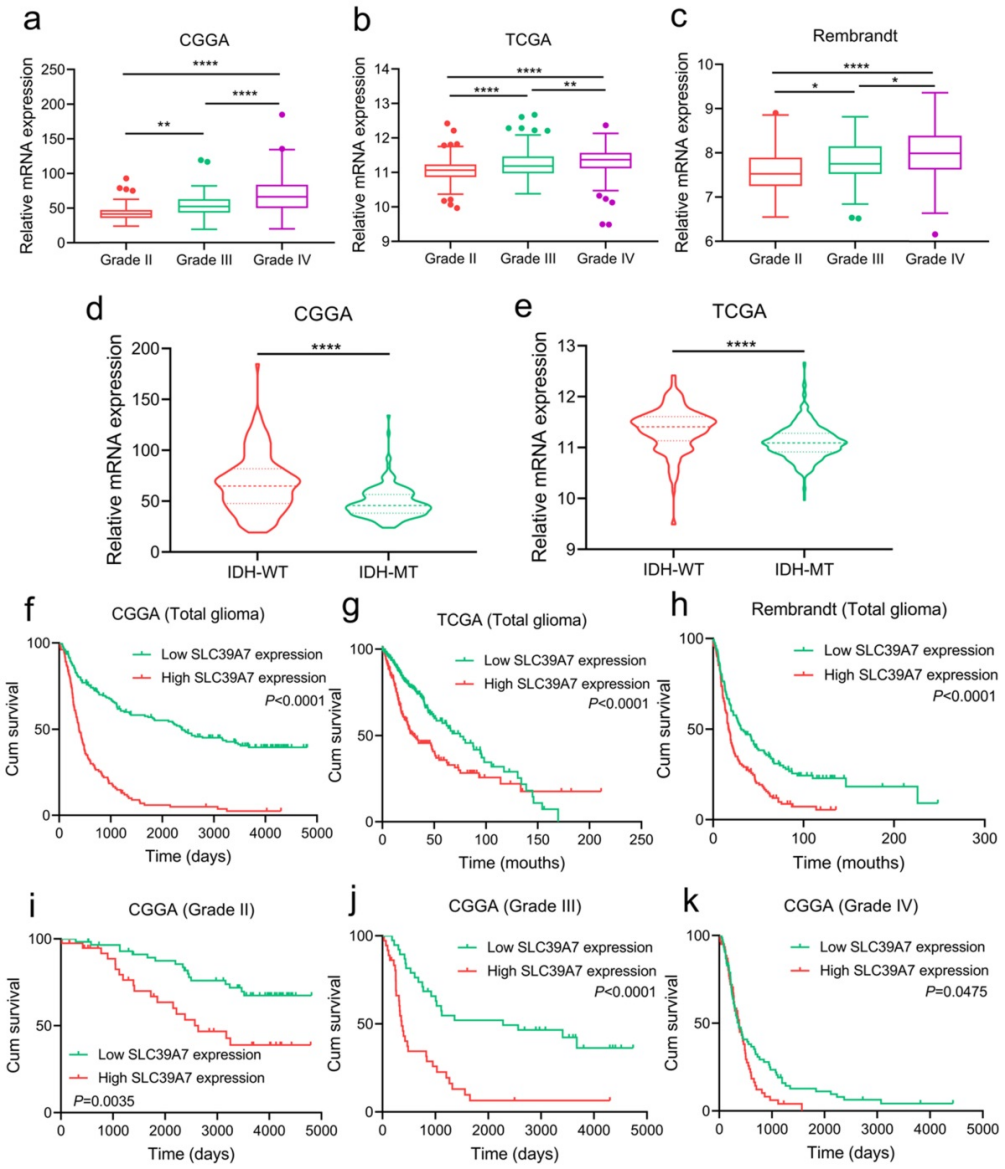
Expression profile and prognostic value of SLC39A7 in glioma databases. a, b, c: The mRNA expression levels of SLC39A7 are shown according to WHO grades in the CGGA (a), TCGA (b) and Rembrandt (c) databases. d, e: The mRNA expression levels of SLC39A7 are shown according to IDH status in the CGGA (d) and TCGA (e) databases. f, g, h: The prognostic significance of SLC39A7 in all glioma patients was detected in the CGGA (f, MST (ranges) of low and high expression of SLC39A7: 2382 (59-4809) days, and 386 (20-4300) days), TCGA (g, MST (ranges): 75 (0.2-169.8) months, and 30.2 (0.2-211.2) months) and Rembrandt (h, MST (ranges): 31.3 (1-248.2) months, and 17.2 (0.3-135.8) months) database. i, j, k: The prognostic significance of SLC39A7 in grade II (i, MST (ranges) of low and high expression of SLC39A7: undefined (529-4809) days, and 2635 (158-4793) days), grade III (j, MST (ranges): 2279 (221-4739) days, and 362 (64-4300) days) and grade IV (k, MST (ranges): 379 (59-4435) days, and 370 (20-1570) days) glioma patients were detected in the CGGA database. All data are shown as the mean ± SD (three independent experiments). *p < 0.05; **p < 0.01; ****p < 0.0001.

To investigate the expression and prognostic value of SLC39A7 in gliomas, we analyzed its expression in CGGA, TCGA and Rembrandt databases. In three databases, we found that SLC39A7 was more highly expressed in WHO grade IV GBM than in WHO grade II or grade III glioma (Fig. 1a-c). To characterize the relationship between SLC39A7 and IDH status, we found that SLC39A7 was highly enriched in IDH wild-type glioma in CGGA and TCGA databases (Fig. 1d, e). Through Kaplan-Meier analysis, SLC39A7 expression was found to be associated with decreased survival rates among all gliomas and different WHO grade gliomas in the CGGA database (Fig. 1f, 1i-k), and high SLC39A7 expression predicted poor survival rates among all glioma in the TCGA and Rembrandt databases (Fig. 1g-h).

**Figure 2:**
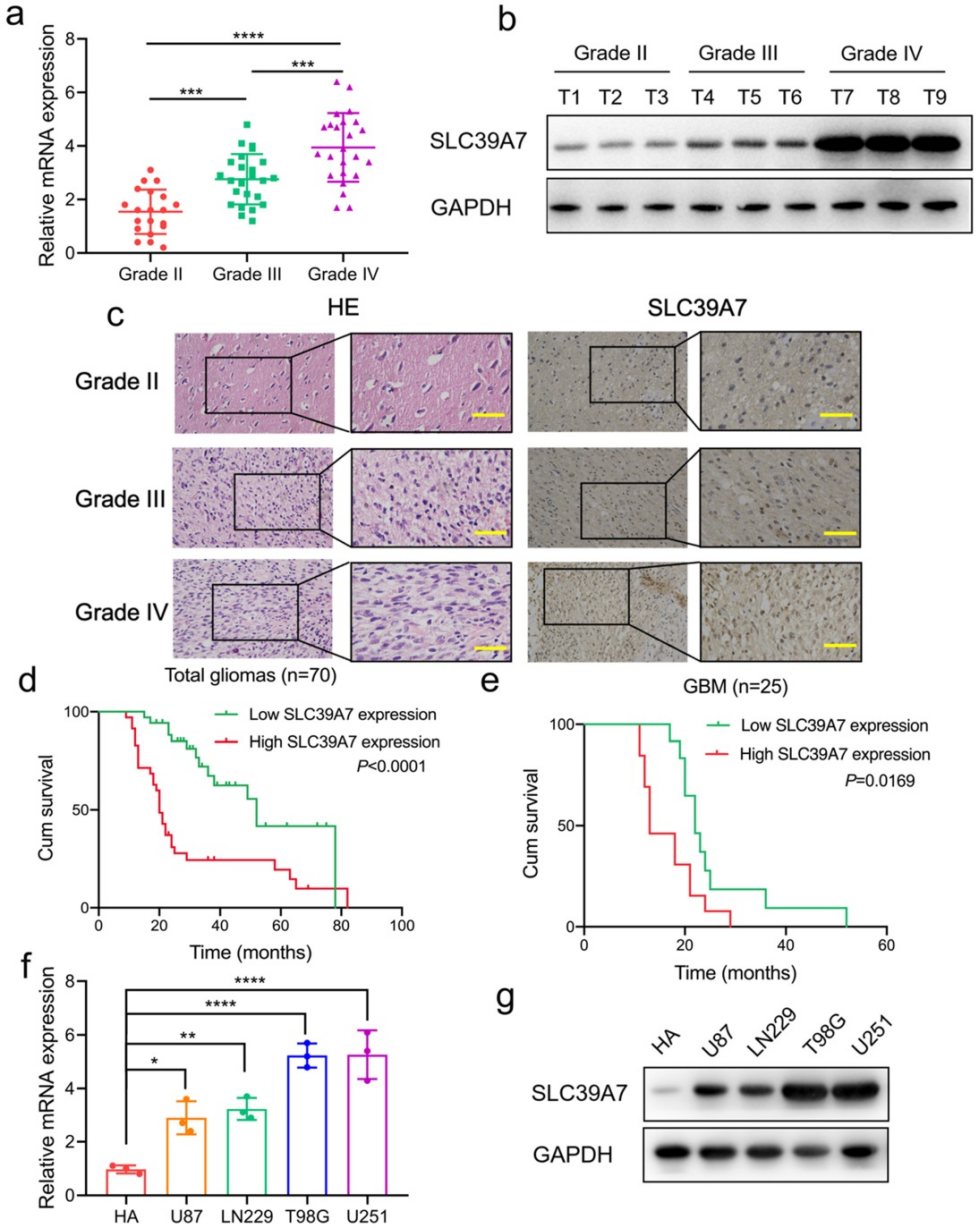
SLC39A7 is highly expressed in glioma and high expression, as detected in clinical samples and glioma cell lines, is related to poor survival. a, b, c: SLC39A7 was expressed at higher levels in different grades of glioma tissue, as detected by qPCR (a), western blotting (b) and IHC (c) (grade II, n = 20; grade III, n = 25; grade IV, n = 25). Scale bar = 50 μm. d, e: Kaplan-Meier analysis showed the prognostic significance of high and low SLC39A7 expression, as detected by qPCR, in 70 glioma patients (d, MST (ranges): 52 (17-78) months, and 20 (11-82) months) and 25 GBM patients (e, MST (ranges): 22 (19-52) months, and 13 (12-29) months). f, g: The expression of SLC39A7 in different glioma cell lines, as measured by qPCR (f) and western blot analysis (g). All data are shown as the mean ± SD (three independent experiments). *p < 0.05; **p < 0.01; ***p < 0.001; ****p < 0.0001.

Then, we investigated the expression of SLC39A7 in our 70 glioma patients. All results, including qPCR, western blotting and IHC, showed that SLC39A7 was highly expressed, and expression was especially increased in higher WHO grade gliomas (Fig. 2a-c). Kaplan-Meier survival analyses showed that the median survival times (MST) of all patients, or GBM patients with higher SLC39A7 expression, were both shorter than in the lower expression group (Fig. 2d, e). IHC and clinical analysis showed that the expression of SLC39A7 was related to WHO grade and IDH status in 70 glioma patients (Table 1). Moreover, both univariate and multivariate cox analyses showed that SLC39A7 expression, IDH status and WHO grades were independent prognostic factors of glioma patients (Table 2). The above results showed that the high expression of SLC39A7 was associated with glioma grade and poor prognosis.

### SLC39A7 regulates the proliferation, invasion and migration of glioma cells

**Figure 3:**
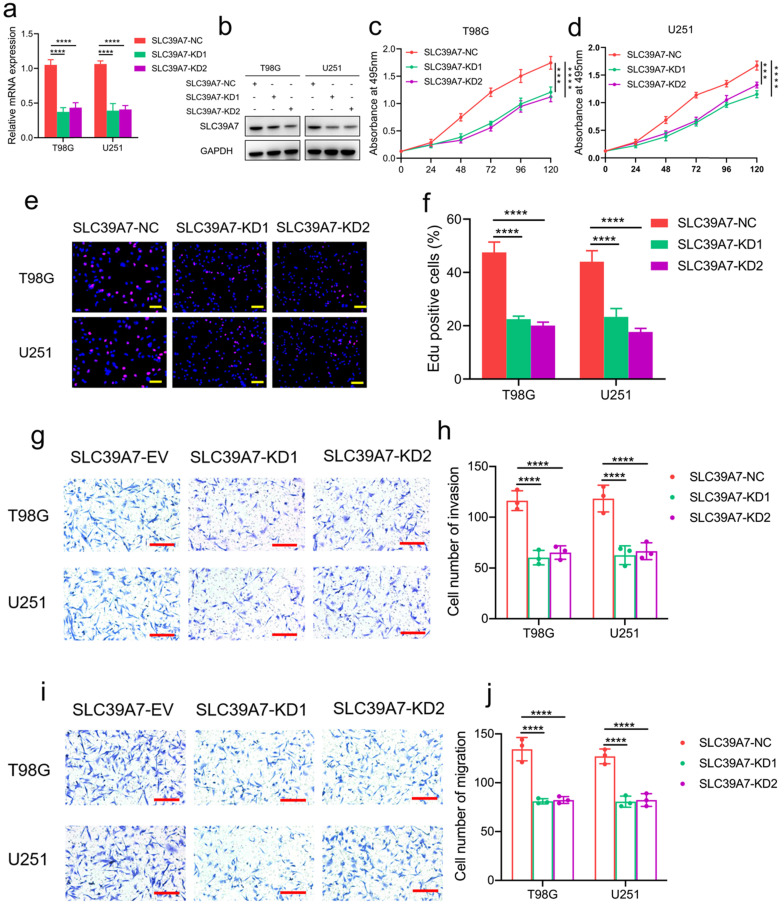
SLC39A7 knockdown inhibited glioma progression *in vitro*. a, b: The effect of SLC39A7 knockdown in T98G and U251 cells, as detected by western blotting (a) and qPCR (b). c, d: MTS assays showing that the T98G and U251 cell viability were decreased after SLC39A7 knockdown. e, f: Edu assay showing that the proliferation of T98G and U251 cells was decreased after SLC39A7 knockdown. Scale bar = 50 μm. g, h: Representative transwell assay showing that the invasion of T98G and U251 cells was decreased after SLC39A7 knockdown. Scale bar = 100 μm. i, j: Representative transwell assay showing that the migration of T98G and U251 cells was decreased after SLC39A7 knockdown. Scale bar = 100 μm. All data are shown as the mean ± SD (three independent experiments). ***p < 0.001; ****p < 0.0001.

To determine the role of SLC39A7 in glioma tumor cells, T98G and U251 glioma cell lines were chosen to stably knockdown the expression of SLC39A7 (Fig. 2f, g). Transfection efficacy was confirmed by western blot analysis and qPCR (Fig. 3a, b). Both MTS and Edu assays confirmed that U251 and T98G cells' proliferation decreased obviously after SLC39A7 knockdown (Fig. 3c-f). The invasion and migration of glioma cells were decreased following SLC39A7 knockdown, as determined by a transwell assay (Fig. 3g-j).

**Figure 4:**
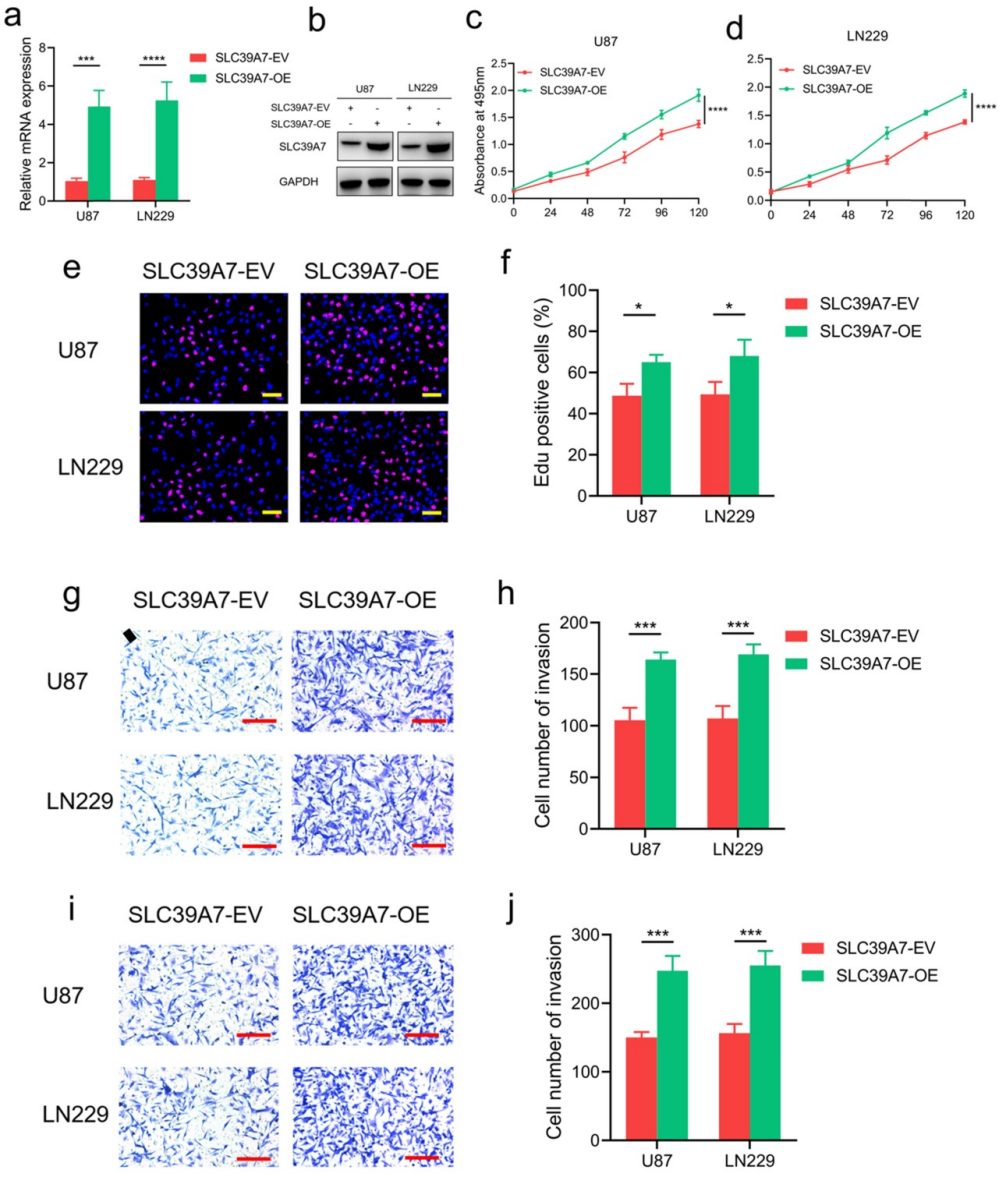
SLC39A7 overexpression promoted glioma progression *in vitro*. a, b: The effect of SLC39A7 overexpression in U87 and LN229 cells, as detected by western blotting (a) and qPCR (b). c, d: MTS assays showing that U87 and LN229 cell viability was increased after SLC39A7 overexpression. e, f: Edu assay showing that the proliferation of U87 and LN229 cells were increased after SLC39A7 overexpression. Scale bar = 50 μm. g, h: Representative transwell assay showing that the invasion of U87 and LN229 cells were increased after SLC39A7 overexpression. Scale bar = 100 μm. i, j: Representative transwell assay showing that the migration of U87 and LN229 cells were increased after SLC39A7 overexpression. Scale bar = 100 μm. All data are shown as the mean ± SD (three independent experiments). *p < 0.05; ***p < 0.001; ****p < 0.0001.

To further characterize the effect of SLC39A7 in glioma cells, we selected the U87 and LN229 cell lines for the overexpression of SLC39A7 since these cell lines had the lowest expression of SLC39A7 among all of the available cell lines (Fig. 2f, g). Western blot analysis and qPCR were performed to confirm the overexpression of SLC39A7 in U87 and LN229 cells (Fig. 4a, b). The results of MTS and Edu assays showed that the proliferation of U87 and LN229 cells were obviously increased after SLC39A7 overexpression (Fig. 4c-f). SLC39A7 overexpression also increased the invasion and migration of these glioma cells compared with the control group (Fig. 4g-j). These results confirmed that SLC39A7 could regulate the proliferation, invasion and migration of glioma cells and that high expression of SLC39A7 promotes the malignant behaviors of glioma cells.

### SLC39A7 promotes glioma tumorigenesis

**Figure 5:**
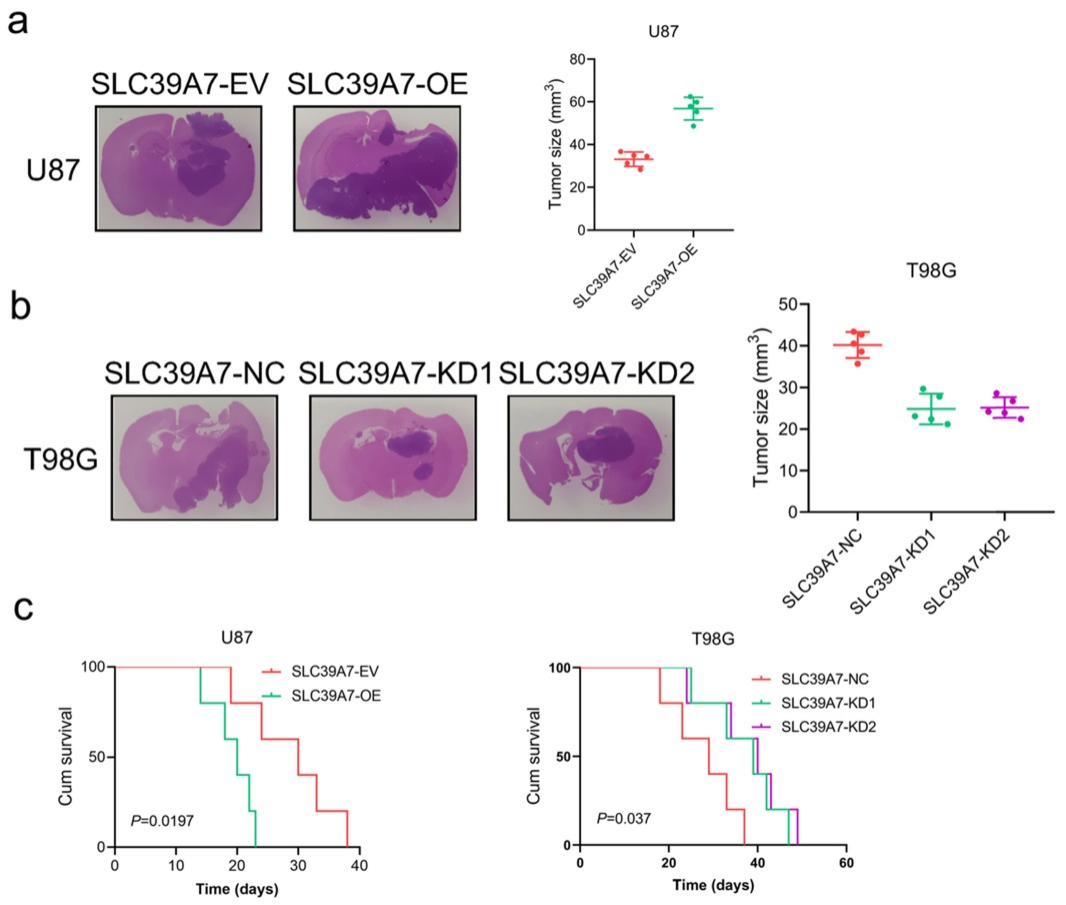
SLC39A7 regulates glioma tumorigenesis. a: Representative photographs showing the intracranial tumor size in the coronal position. Scale bar =10 mm. b: Kaplan-Meier survival curves showing the survival times of the SLC39A7 overexpression group (MST (ranges): 30 (24-38) days of SLC39A7 empty vector group, and 20 (18-23) days of SLC39A7 overexpression group, n = 5). c: Kaplan-Meier survival curves showing the survival times of the SLC39A7 knockdown group (MST (ranges): 29 (23-37) days of SLC39A7 natural control group, 39 (33-47) days of SLC39A7 knockdown 1 group, and 40 (34-49) days of SLC39A7 knockdown 2 group, n = 5). All data are shown as the mean ± SD (three independent experiments). *p < 0.05; **p < 0.01; ***p < 0.001.

We further evaluated the effects of SLC39A7 on glioma tumorigenesis by orthotopic xenograft models. We found that the intracranial tumor volume was significantly decreased after SLC39A7 overexpression in U87 cells. According to Kaplan-Meier survival analysis, the MST of the U87-SLC39A7 EV group was 30 days. The overexpression of SLC39A7 decreased the MST to 20 days (Fig. 5a, c). Knockdown of SLC39A7 in T98G significantly extended the MST of nude mice to 39 and 40 days (MST, control group = 29 days), which was consistent with the significant smaller tumor volumes observed in the SLC39A7 knockdown group compared with the control group (Fig. 5b, c). Taken together, these results clearly suggested that SLC39A7 plays a significant role in promoting tumorigenesis in nude mice.

### SLC39A7 promotes the malignant behaviors of glioma by activating the TNF-α-mediated NF-κB signaling pathway

**Figure 6:**
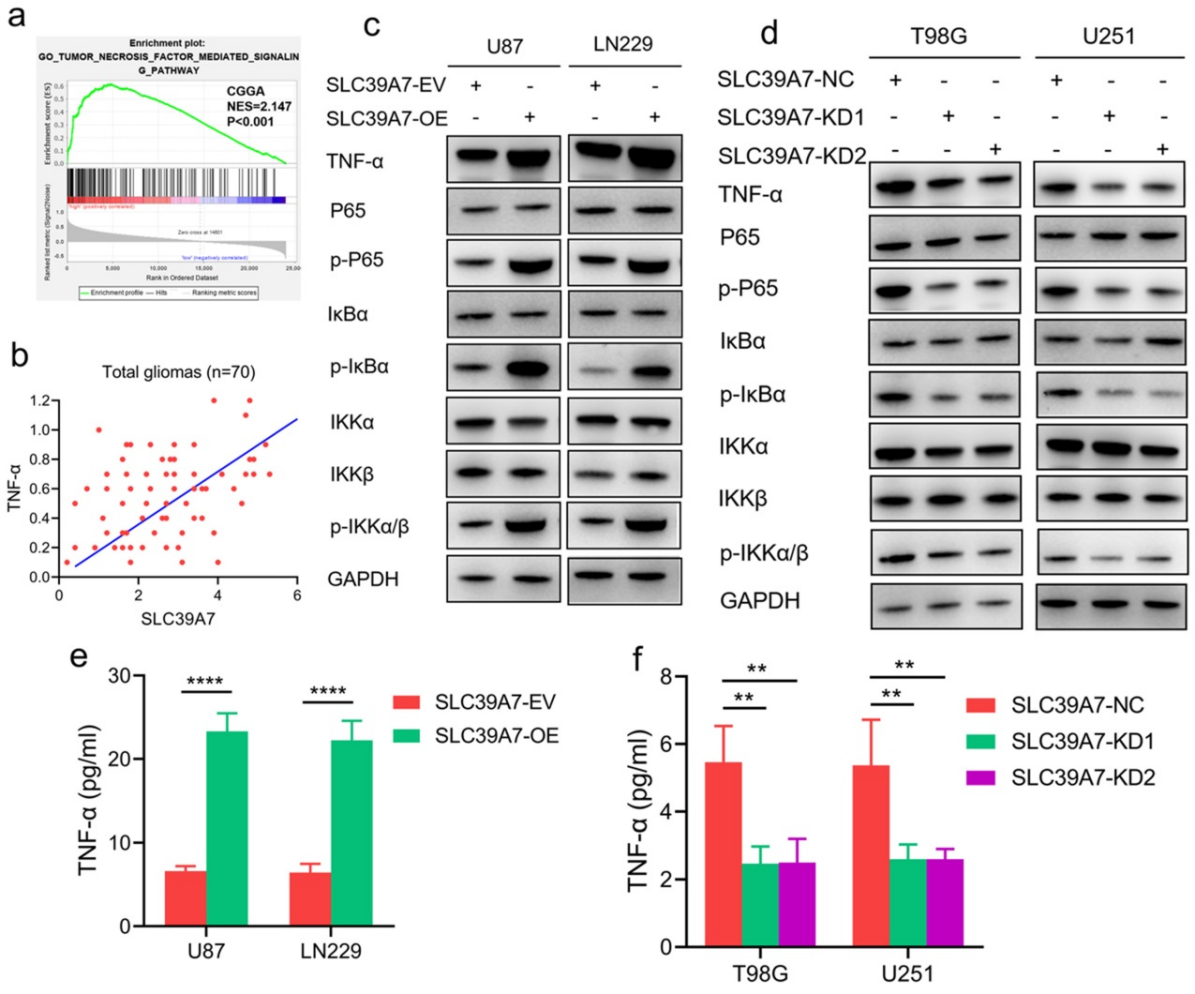
SLC39A7 activates the TNF-α-mediated NF-κB signaling pathway. a. GSEA results based on SLC39A7 expression in the CGGA database. b. The correlation between SLC39A7 and TNF-α in the clinical samples. c. The expression of TNF-α and NF-κB downstream targets after SLC39A7 overexpression, as measured by western blotting. d. The expression of TNF-α and NF-κB downstream targets after SLC39A7 knockdown, as measured by western blotting. e, f: The secretion of TNF-α after SLC39A7 overexpression (e) or knockdown (f), as measured by ELISA. All data are shown as the mean ± SD (three independent experiments). **p < 0.01; ****p < 0.0001.

**Figure 7:**
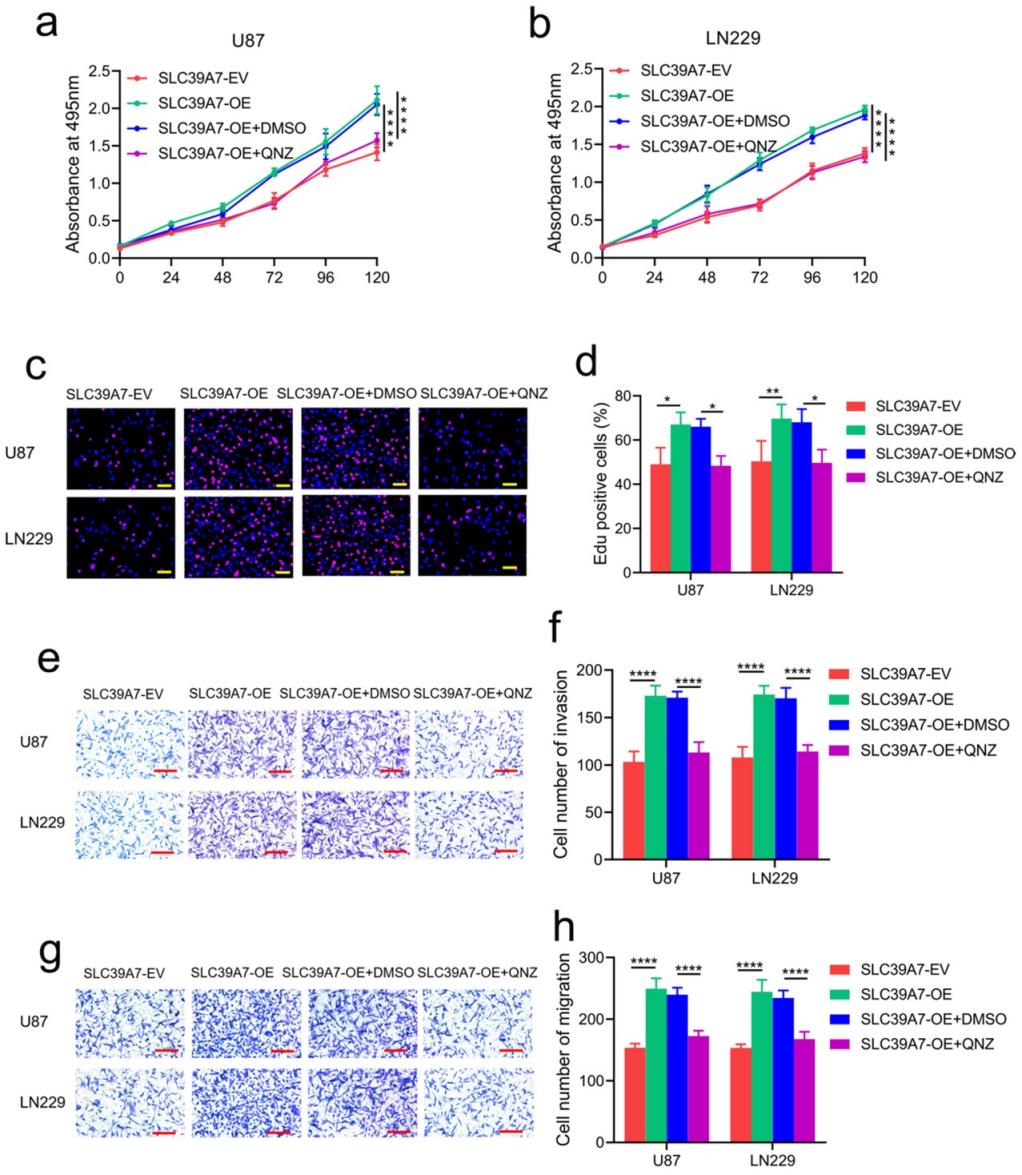
TNF-α inhibitor abrogated the promotion effects of SLC39A7. a, b: MTS assays showing that the increased cell viability induced by SLC39A7 overexpression was reversed after QNZ treatment. c, d: Edu assay showing that the increased proliferation induced by SLC39A7 overexpression was reversed after QNZ treatment. Scale bar = 50 μm. e, f: Representative transwell assay showing that the increased invasion induced by SLC39A7 overexpression was reversed after QNZ treatment. Scale bar = 100 μm. g, h: Representative transwell assay showing that the increased migration induced by SLC39A7 overexpression was reversed after QNZ treatment. Scale bar = 100 μm. All data are shown as the mean ± SD (three independent experiments). *p < 0.05; **p < 0.01; ***p < 0.001.

To determine the specific signaling pathway of SLC39A7 involved in glioma, we performed GSEA based on the CGGA databases. We found that high SLC39A7 expression is related to the TNF-α-mediated signaling pathway (Fig. 6a). Furthermore, we detected the mRNA expression levels of SLC39A7 and TNF-α in 70 clinical glioma specimens. The results showed a significant positive correlation between SLC39A7 and TNF-α expression in all patients (r=0.4328, *P*=0.0002, Fig. 6b). Since our previous study showed that TNF-α could promote proliferation and tumorigenesis via the NF-κB signaling pathway in glioma, we then detected downstream molecules' expression. As determined by western blot analysis, the expression levels of TNF-α, p-P65, p-IκBα and p-IKKα/β were all significantly increased following SLC39A7 overexpression in U87 and LN229 cells (Fig. 6c). Conversely, the above molecules' expression was significantly downregulated after SLC39A7 knockdown in T98G and U251 cells (Fig. 6d). Then, ELISA revealed that the secretion of TNF-α was increased after SLC39A7 overexpression and decreased after SLC39A7 knockdown (Fig. 6e, f). To further confirm whether SLC39A7 promotes the proliferation, invasion and migration of glioma cells by activating TNF-α, SLC39A7-overexpressing U87 and LN229 cells were treated with QNZ (EVP4593), a TNF-α inhibitor. Both MTS and Edu assays indicated that the increased proliferation caused by SLC39A7 overexpression was reversed by QNZ treatment (Fig. 7a-d). A transwell assay showed that the increased invasion and migration of SLC39A7-overexpressing glioma cells were reversed by QNZ treatment (Fig. 7e-h). These data suggested that SLC39A7 may promote glioma proliferation, invasion and migration via upregulation of the expression and secretion of TNF-α and activation of the NF-κB signaling pathway.

## Discussion

As the most malignant intracranial tumor, the clinical treatment of glioma has encountered great challenges 20. Current treatment methods have not achieved satisfactory results, and further studies are needed to screen for pathogenic genes and study pathogenic mechanisms to provide new insight into treatment strategies for glioma. This study revealed a novel gene, SLC39A7, that is highly expressed in glioma tissues and cells. Through analysis of the TCGA, CGGA and Rembrandt databases, we found that SLC39A7 was expressed more highly in GBM than in other grades of glioma. Furthermore, Kaplan-Meier survival analysis revealed that the patients with high SLC39A7 expression had a worse prognosis. These findings indicated that SLC39A7 might play a vital role in glioma progression.

SLC39A7 is a member of the zinc transporter solute carrier family 39 (SLC39), which transports zinc into the cytosol from the extracellular space or intracellular stores 21, 22. SLC39A7 was reported to decrease cytosolic zinc levels, increased ER zinc levels, impaired cell proliferation and the induction of ER stress 23. A recent study indicated that SLC39A7 deficiency could lead to reducing B-cell receptor signaling strength and positive selection 24. Furthermore, phosphorylation and/or overexpression of SLC39A7 in the ER results in increasing cytosolic zinc concentrations, stimulating glucose uptake, which can cause insulin resistance and type 2 diabetes mellitus 25.

SLC39A7 has also been reported to be an important oncogene in several cancers. Increased levels of zinc and SLC39A7 were found in tamoxifen-resistant breast cancer MCF-7 cells 26. In cervical cancer, SLC39A7 promotes cell proliferation, migration and invasion, and reduces apoptosis through downregulation of Bax and E-cadherin and upregulation of Bcl-2 and MMP-2 14. Moreover, knockdown of SLC39A7 inhibited the growth of colorectal cancer cells through G2/M cell cycle arrest and apoptosis 27. However, it is not clear whether SLC39A7 participates in the tumorigenesis and development of gliomas.

In our study, the results of MTS, EdU and transwell assays indicated that SLC39A7 overexpression promoted the proliferation, invasion and migration of U87 and LN229 cells, while knockdown of SLC39A7 led to the opposite result. In orthotopic xenograft models, knockdown of SLC39A7 inhibited the glioma volume and increased nude mice's survival time. Taken together, these results indicated that the high expression of SLC39A7 mediated an oncogenic effect in glioma.

To further confirm the mechanism of action of SLC39A7 in glioma, we conducted GSEA on the CGGA database. We found that high SLC39A7 expression enriched positive regulation of the TNF-α signaling pathway. Several studies have shown that TNF-α promotes cell proliferation and migration, treatment resistance, and the induction of apoptosis by activating the NF-κB signaling pathway 28-30. A recent study indicated that knockdown of SLC39A7 led to necroptosis resistance by affecting TNF receptor surface levels 31. Our results showed that SLC39A7 overexpression increased the expression and secretion of TNF-α, which upregulated the expression of the main molecules involved in the NF-κB signaling pathway, such as p-P65, p-IκBα and pIKKα/β. Rescue experiments involving QNZ treatment of SLC39A7-overexpressing glioma cells confirmed that the promotion effects of SLC39A7 were abrogated. Therefore, SLC39A7 may activate the TNF-α-mediated NF-κB signaling pathway, thereby promoting the malignant progression of glioma.

## Conclusion

In summary, we found that the zinc transporter SLC39A7 is highly expressed in high-grade glioma patients with a poor prognosis and can activate the TNF-α-mediated NF-κB signaling pathway, thereby promoting the proliferation, invasion and migration of glioma cells. SLC39A7 may, therefore, be a therapeutic target to inhibit glioma progression and improve the prognosis of patients.

## Figures and Tables

**Table 1:** Relationship of SLC39A7 expression to clinical features of glioma patients.

Clinical features		Samples (*n* = 70)	SLC39A7 expression*		*P* value
			Low (*n* = 29)	High (*n* = 41)	
**Sex**	Male	36	14	12	P=0.5221
	Female	34	15	18	
**Age**	≤ 50	31	11	20	P=0.3680
	> 50	39	18	21	
**IDH status**	Wild	42	10	32	**P=0.0002**
	Mutant	28	19	9	
**WHO grade**	II	20	15	5	**P<0.0001**
	III	25	9	16	
	IV	25	5	20	

*: SLC39A7 expression was detected by immunohistochemistry and evaluated according to the German immunohistochemical score. High expression was defined as score ≥4.

**Table 2:** Univariate and multivariate cox analysis of glioma patients.

Factors	Categories	Univariate analysis			Multivariate analysis	
		Χ^2^	P value	HR	95% CI	P value
**Gender**	Male/female	1.9916	0.1582	0.8940	0.462-1.729	0.7392
**Age**	≤50/>50	0.1099	0.7402	1.0403	0.530-2.042	0.9086
**WHO grade**	Grade II	39.9786	**<0.0001**	1.9000	2.262-18.389	**0.0469**
	Grade III					
	Grade IV					
**IDH status**	Mutant/wild	32.5198	**<0.0001**	6.4491	1.009-3.578	**0.0004**
**SLC39A7**	High/low	15.575	**<0.0001**	2.4664	1.189-5.116	**0.0153**
